# Sinomenine Suppresses Osteoclast Formation and *Mycobacterium tuberculosis* H37Ra-Induced Bone Loss by Modulating RANKL Signaling Pathways

**DOI:** 10.1371/journal.pone.0074274

**Published:** 2013-09-16

**Authors:** Xiaojuan Li, Longgang He, Yiping Hu, Heng Duan, Xianglian Li, Suiyi Tan, Min Zou, Chunping Gu, Xiangzhou Zeng, Le Yu, Jiake Xu, Shuwen Liu

**Affiliations:** 1 School of Pharmaceutical Sciences, Southern Medical University, Guangzhou, China; 2 School of Pathology and Laboratory Medicine, University of Western Australia, Crawley, Western Australia, Australia; University of Muenster, Germany

## Abstract

Receptor activator of NF-κB ligand (RANKL) is essential for osteoclastogenesis. Targeting RANKL signaling pathways has been an encouraging strategy for treating lytic bone diseases such as osteoporosis and rheumatoid arthritis (RA). Sinomenine (SIN), derived from Chinese medicinal plant *Sinomenioum* *acutum* , is an active compound to treat RA, but its effect on osteoclasts has been hitherto unknown. In the present study, SIN was found to ameliorate *M. tuberculosis* H37Ra (Mt)-induced bone loss in rats with a decreased serum level of TRACP5b and RANKL, and an increased level of osteoprotegerin (OPG). *In vitro* study also showed that SIN could inhibit RANKL-induced osteoclast formation and bone resorption. The osteoclastic specific marker genes induced by RANKL including c-Src, MMP-9, TRACP were inhibited by SIN in a dose dependent manner. Signal transduction studies showed that SIN could obviously reduce the expression of RANK adaptor molecule TRAF6 and down-regulate RANKL-induced NF-κB activation. It decreased the RANKL-induced p38, JNK posphorylation but not ERK1/2 posphorylation. SIN could also reduce RANKL-mediated calcium influx which is associated with TRAF6/c-Src complex. Finally, SIN suppressed RANKL induced AP-1 and NFAT transcription, as well as the gene expression of NFATc1 and AP-1 components (Fra-1, Fra-2, c-Fos). The protein expression of c-Fos and TRAF6 were also inhibited by SIN after RANKL stimulation. Taken together, SIN could attenuate osteoclast formation and Mt-induced bone loss by mediating RANKL signaling pathways.

## Introduction

Bone remodeling depends on a delicate balance between bone formation and bone resorption [1,2]. Increased bone resorption is a hallmark of several lytic bone diseases such as rheumatoid arthritis (RA), psoriatic arthritis and osteoporosis, due to the elevated activity of osteoclasts [3-5]. Osteoclasts are bone resorptive multinucleated cells originating from monocyte/macrophage linage [6]. In RA patients, osteoclasts were identified in bone marrow, synovial pannus and/or bone resorption lacunae [7].

Differentiation of osteoclasts from the precursors is regulated by soluble and membrane-bound molecules supported by osteoblasts and other cells in the bone microenvironments [8]. Receptor activator of nuclear factor-κB ligand (RANKL) plays an essential role in osteoclastogenesis. The RANKL knockout mice are deficient of osteoclasts [9]. Also, the blockade of RANKL binding to its receptor or genetic absence of RANKL can abrogate joint damage despite the presence of joint inflammation in the arthritis animals [10]. In RA patients, RANKL expression was demonstrated in B cells of synovial fluid [11], synovial fibroblasts and activated T cells [7,12]. Denosumab, an anti-RANKL monoclonal antibody approved by FDA for treating diseases with increased bone resorption [13] and osteoclastogenesis [14], targets RANKL directly. It can alleviate the joint erosion in RA patients [15]. RANKL signaling pathway thus holds promise as a therapeutic target for suppressing excessive osteoclasts formation in a variety of lytic bone diseases [6].

The sequential molecular events induced by RANKL during osteoclast differentiation are summarized in a review by Asagiri M, et al [6]. Firstly, RANKL binds to its receptor RANK that recruits adaptor molecules such as TRAF6 (Tumor necrosis factor receptor associated factor 6). The binding of TRAF6 to RANK results in the activation of NF-κB and mitogen-activated protein kinases (MAPKs). NF-κB (Nuclear factor-kappa B) is among the very early molecular events induced by RANKL, MAPKs are involved in the activation of AP-1 (Activator protein-1) components. NF-κB and AP-1 are important for the initial induction or autoamplification of NFATc1 (Nuclear factor of activated T-cells cytoplasmic 1) which is activated by calcium signaling. Finally, in cooperation with other transcription factors, NFATc1 activates the osteoclast-specific genes such as TRACP (Tartrate-resistant acid phosphatase), Cathepsin K, CTR (calcitonin receptor), MMP-9 (Matrix metalloproteinase 9), etc and induces osteoclastogenesis. In addition, osteoprotegerin (OPG), which is regarded as the decoy receptor for RANKL, attenuates excessive RANKL signaling [16].

Sinomenine (SIN), with the chemical name 7,8-didehydro-4-hydroxy-3,7- dimethoxy-17-methyl-morphinane-6-one, is an alkaloid isolated from the Chinese medicinal plant, *Sinomenium* *acutum* Rehder *& Wilson*, which has been used to treat rheumatic and arthritic diseases for over 1000 years in China and Japan [17]. SIN has significant analgesic, anti-arthritic, anti-inﬂammatory and immunosuppressive properties [18]. SIN could significantly improve adjuvant or collagen-induced arthritis by inhibiting synovial fibroblasts [19], modulating MMPs/TIMPs (Tissue inhibitors of metalloproteinase) and cytokines [20], suppressing anti-CII (Type II collagen) antibodies and Th1/Th2 responses [21]. SIN could also reduce the invasion and migration in co-cultured fibroblast-like synoviocytes and human THP-1 cells by inhibiting MMP-2, MMP-9 and CD147 expression [22], suppress the IL-1β-induced genes expression in a human synovial sarcoma cell line [23], as well as protect IL-1β-induced proteoglycan degradation and apoptosis in rabbit articular cartilage and chondrocyte [24]. In addition to these pharmaceutical effects, Zheng Qing Feng Tong Ning (ZQFTN), the clinical drug made of purified hydrochloric sinomenine, has been shown to have therapeutic efficacy and low side effects in RA patients in China since 1990s [18]. Besides, the enteric-coated tablets, sustained release tablets and the injection of ZQFTN, are all recorded in the Chinese National Pharmacopoeia to treat RA. However, the role of SIN in osteoclast formation, bone resorption and its mechanism of action has been hitherto unknown.

*M. tuberculosis* H37Ra (Mt)-induced autoimmune arthritis in Lewis rats is an RA model to investigate drug effects on bone damage [25]. We also observed an obvious bone loss around ankle joint and increased serum RANKL, RANKL/OPG ratio, osteoclast activity in Mt-induced arthritis in SD rats. In this study, SIN was found to reduce Mt-induced bone damage, osteoclast activity and RANKL signals *in vivo* first. Then SIN was observed to exhibit inhibitory effects on RANKL induced osteoclast differentiation, bone resorption and osteoclastogenesis related signaling pathways in murine pre-osteoclastic cells *in vitro*. Our results suggested that SIN could attenuate RANKL signaling both *in vitro* and *in vivo*, resulting in the suppression of osteoclasts formation and bone loss.

## Materials and Methods

### Materials

Sinomenine (SIN, >98% purity by HPLC) was purchased from Ronghe Inc. (Shanghai, China). Soluble recombinant RANKL (Cat. 462-TEC) was obtained from R&D Inc. (Lorton, VA). Leukocyte acid phosphoatase assay Kit (Cat. 387-A) and 4,6-diamidino-2-phenylindole (DAPI) were obtained from Sigma-Aldrich (St Louis, MO). Dulbecco’s modified Eagle’s medium (DMEM), α-modification of eagles medium (α-MEM), fetal bovine serum (FBS), penicillin, streptomycin and Alexa Fluor 488 labeled goat anti-rabbit secondary antibody were purchased from Invitrogen (San Diego, CA). Cell counting kit-8 (CCK-8) was obtained from Dojindo Molecular Technologies (Japan). PrimeScript RT reagent kit and SYBR Premix Ex Taq were purchased from TaKaRa (Dalian, China). The luciferase reagents and the CellTiterGlo luminescent reagents were purchased from Promega (Madison, WI). Turbofect in vitro transfection reagent was obtained from Fermentas. Nuclear Extraction Kit was purchased from Cayman Chemical (Ann Arbor, Michigan). TRAF6 antibody was obtained from Epitomics, Inc. (Burlingame, CA), and other antibodies were obtained from Cell Signaling Technology (Danvers, MA). Rat RANKL, OPG and TRACP5b ELISA Kits were purchased from Westang Biotech Co. Ltd (Shanghai, China). Osteo Assay plates were obtained from Corning Life Science (St. Lowell, MA). NFAT-TA-luc and AP-1-luc plasmids were purchased from Clontech Company(Mountain View, CA). Renilla luciferase control vector was obtained from Promega (Madison, WI). NF-κB-luc stably tranfected RAW264.7 cells were described previously [26].

### Animals

Female Sprague-Dawley (SD) rats (180-200 g, 7-8 Weeks) and BALB/c mice were obtained from the Animal Breeding Center of the Southern Medical University. The animal experiments were approved by the Institutional Animal Care and Use Committee of the Southern Medical University. The animals were maintained in a well-ventilated controlled room at 25°C on a 12-h light/dark cycle with free access to water and food. Mice were anesthetized by diethyl ether and bled before collecting bone marrow cells. Rats were anesthetized by 10% chloral hydrate before bleeding at day 28, and all efforts were made to minimize suffering.

### Evaluation of bone loss in Mt-induced arthritis

Arthritis was induced in SD rats by injecting subcutaneously at the base of the tail with 200 µl of heat-killed *M. tuberculosis* H37Ra (Mt) (Difco, Detroit, MC) at 2 mg/rat in mineral oil [27]. Control rats were not immunized with Mt emulsion. SIN was then administrated intraperitoneally at a dosage of 80 mg/kg/day. *Tripterygium* *wilfordii* polyglycosides (TWP), the anti-arthritis Chinese drug (Forward Pharmaceutical Company, Shanghai, China), was fed at 2.5 mg/kg/day. All animals were examined regularly on hind paw edema indicated by the volume. After 28 days, blood was taken from rat abdominal aorta after anesthesia and then serum was prepared and stocked to measure RANKL, OPG and TRACP5b level by ELISA kits (Westang, Shanghai, China). The alkaline phosphatase (ALP) activity test kit (Jiancheng Bioengineering Institute, Nanjing, China) was used to detect serum ALP activity for osteoblast activity. After the rats were killed, the hind paws were dissected and the specimen was prepared as described before [27] to detect the histological damage and osteoclasts of arthritic ankle joints by hematoxylin-eosin (HE) and TRACP (Sigma-Aldrich) staining respectively; the hind limbs of another side were fixed and kept in 10% formalin for analysis of bone erosion by a micro-CT scanner (ZKKS-MCT-Sharp, Guangzhou, China). To achieve bone parameters by micro-CT, the hind paws (from the tip of the toes to the middle of the tibia) obtained from experimental rats were scanned and reconstructed into a three-dimensional structure with a voxel size of 18 µm. The X-ray tube was operated at a voltage of 50 kV, power of 40 W, and exposure time of 300 ms through a 0.5-mm-thick aluminum filter. The X-ray projections were obtained at 0.72° intervals with a scanning angular rotation of 360°. The reconstructed dataset was segmented by an automated thresholding algorithm. The projection images were reconstructed into three-dimensional images using ZKKS-MicroCT software (version 4.1) from ZKKS.

### *In vitro* osteoclastogenesis TRACP assay

Bone marrow macrophages (BMM) were isolated from the long bones of BALB/c mice and seeded into a 96-well plate (2.5×10^5^ cells per well) in the presence of 50 ng/ml of macrophage-colony stimulating factor (M-CSF) and 50 ng/ml RANKL for 7 days. SIN was administered and the medium was replenished at day 3. The tartrate-resistant acid phosphatase (TRACP) positive multinucleated cells (>3 nuclei) were counted as osteoclasts by using Leukocyte Acid Phosphoatase Assay Kit (Sigma-Aldrich) [28].

RANKL can efficiently induce osteoclast formation in RAW264.7 cells. To study the effects of SIN on RANKL signaling *in vitro*, we chose RAW264.7 cells as cell culture model of osteoclast differentiation. For TRACP assay, RAW264.7 cells were cultured in α-MEM supplemented with 10% FBS and 100 U/ml penicillin/streptomycin with a density of 2×10^3^ cells per well. Cells were incubated overnight in a humidified incubator (37 °C, 5% CO_2_) and then stimulated with RANKL (100 ng/ml) alone, or in the presence of indicated concentrations of SIN. Medium was replenished at day 3. After 5 days’ culture, cells were fixed and stained for TRACP. TRACP positive multinucleated cells(>3 nuclei) were counted as osteoclasts [29]. The cell viability of SIN on RAW264.7 cells were evaluated by using CCK-8 assay. In brief, 1×10^4^ cells per well were seeded into 96-well plates for 24 h and then cultured with SIN for 48 h or 7 days. The optical density value after incubating with CCK-8 was measured by microplate reader (GENios Pro, TECAN, Switzerland).

### Bone resorption pit assay

RAW264.7 cells were suspended in α-MEM with 10% FBS and 100 ng/ml RANKL and plated on a 96-well Corning Osteo Assay plate at an initial density of 1×10^3^ cells/well. Various concentrations of SIN (0.5 and 1 mM) were administered at day 4. At day 7, medium was aspirated completely from the wells and the cells were removed using double-distilled water. After wiping, the plates were stained with hematoxylin for 2 min followed by extensive washing in double-distilled water [33]. Images and numbers of the resorbed pits in each well were collected with a light microscope (Nikon, Tokyo, Japan). The area of resorption was analyzed by Image-Pro Plus software (Media Cybernetics, Rockville, MD).

### Luciferase reporter gene assays of NF-κB, NFAT and AP-1

To examine NF-κB activation, both stable and transient NF-κB luciferase reporter genes systems were employed on RAW264.7 cells [28,30]. Firstly, RAW264.7 cells that stably transfected with a NF-κB luciferase reporter gene were used and plated in 96-well plates at a density of 1×10^4^ cells/well and pre-treated with SIN for 30 min followed by 100 ng/ml RANKL stimulation for 8 h. For transient NF-κB reporter gene expression, RAW264.7 cells were plated in 96-well plates at a density of 5×10^4^ cells/well and transfected with NF-κB-luc plasmid by Lipofectin 2000 (Invitrogen) and then treated with RANKL and SIN for 8 h. At the end of the culture, cells were lysed and the luciferase activity was measured by the Luciferase Assay System according the manufacturer’s instructions (Promega).

To examine AP-1 or NFAT activation, RAW264.7 cells were seeded in 96-well plates at a density of 1×10^4^ cells/well and incubated for 24 h. The cells were then transfected with 0.2 μg of the AP-1 or NFAT luciferase reporter constructs (AP-1-luc and NFAT-luc, respectively) using Turbofect (Fermentas) according to the manufacturer’s instructions. After 48 h, medium was changed with complete DMEM and pre-treated with SIN for 30 min followed by adding RANKL (100 ng/ml) for 12 h, the cells were then harvested and measured for luciferase activity using the Luciferase Assay System.

The transient NF-κB, AP-1 or NFAT luciferase activities were also tested by the Dual-Luciferase Reporter Assay System (Promega) to normalize the transfection efficiency with Renilla luciferase activity (Figure S3).

### P65 nuclear translocation and calcium influx measured by confocal microscopy

For p65 translocation study, RAW264.7 cells were seeded in 24-well plates and incubated overnight. Cells were then pretreated with SIN (1 mM) for 30 min prior to the 15 min stimulation of RANKL (100 ng/ml), fixed with 4% (v/v) paraformaldehyde for 15 min at room temperature, rinsed three times with PBS, and made permeable using ice-cold 100% methanol for 10 min in a freezer. The cells were washed and blocked with 10% (v/v) normal goat serum (Invitrogen) for 1 h at room temperature. The cells were then incubated with primary rabbit anti-mouse NF-κB p65 antibody (1:200, Cell signaling) at 4 °C overnight followed by adding Alexa Fluor 488 labeled goat anti-rabbit secondary antibody (1:500, Invitrogen) for 2 h at room temperature. Finally, DAPI was added to cells for 2 min. Fluorescent signals were detected using a confocal microscopy (FluoView FV1000, Olympus, Japan).

For Ca^2+^ measurement, RAW264.7 cells were incubated with RANKL (100 ng/ml) in the presence or absence of SIN (0.5 mM) for 72 h. Cells were then incubated for 30 min in the presence of 5 μM fluo-3 AM and 0.05% pluronic F127 in serum-free DMEM. After washing twice with DMEM, the cells were incubated in DMEM containing 10% FBS for 20 min, then washed three times with Hank's balanced salt solution and mounted on a confocal microscopy (FluoView FV1000, Olympus, Japan). Cells were excited at 488 nm and emissions at 505–530 nm were acquired simultaneously at 20 s intervals. To estimate intracellular Ca^2+^ concentration fluorescence image and intensity of the fluo-3/ calcium were recorded within 200s.

### Western blot analysis for testing p65, IκBα, TRAF6, c-Fos and MAPKs

RAW264.7 cells (2×10^7^) were seeded in 60 mm petri dish and incubated for 24 h. Cells were then pretreated with SIN (0.25, 0.50 and 1 mM) for 30 min followed by 30 min RANKL (100 ng/ml) stimulation. Whole cell extracts were prepared using Buffer A (pH 7.5, containing 50 mM Tris-HCl, 150 μM sodium chloride, 0.5% cholate acid, 0.1% SDS, 2 mM EDTA, 1% Triton, and 10% glycerol) with protease and phosphatase inhibitors (1 mmol/L phenylmethylsulfonyl fluoride, 1 mmol/L Na _3_VO_4_, and 1 mmol/L NaF). Nuclear and cytoplasmic proteins were prepared using Nuclear Extraction Kit (Cayman) according to the manufacturer’s protocol. Samples (20–50 µg protein) were loaded on 10% SDS-PAGE and transferred to polyvinylidene diﬂuoride membranes. After blocking with 5% nonfat milk in TBST (containing 0.1% Tween 20) for 2 h at room temperature, the membranes were incubated with a primary antibody (1:1000, Cell signaling) and then a conjugated secondary antibody (1:2000,Cell signaling). PDI, Lamin B1 (cell signaling) were employed as antibodies specific for purely cytoplasmic proteins or purely nuclear proteins respectively in certain experiments. The membrane was incubated with ECL detection reagent and then exposed to X-ray ﬁlm (Kodak). The films were scanned and the proteins were quantified by Quantity One software (Bio-Rad, US).

### RNA isolation and Real time PCR

RAW264.7 cells at 2×10^6^ /well were seeded into 6-well plates and then incubated with RANKL (100 ng/mL) and SIN at indicated concentrations (0.25, 0.50 and 1 mM) for 24 h. Total RNA was extracted using Trizol reagent (Invitrogen), and reverse-transcribed using PrimeScript RT reagent kit (TaKaRa) to obtain cDNA. Real-time PCR was performed in an ABI7500 real-time PCR instrument (Applied Biosystems) with the SYBR Premix Ex Taq (TaKaRa). Relative Quantiﬁcation (RQ) was used to determine the fold-change of gene expression compared to GAPDH control [31].

### Statistical Analysis

The results are shown as mean ± SEM. ANOVA was used to test statistical significance between groups. In all cases, a P value of <0.05 was considered to be statistically significant.

## Results

### Sinomenine ameliorated bone loss and osteoclast activity in Mt induced arthritic rats

**Figure 1:**
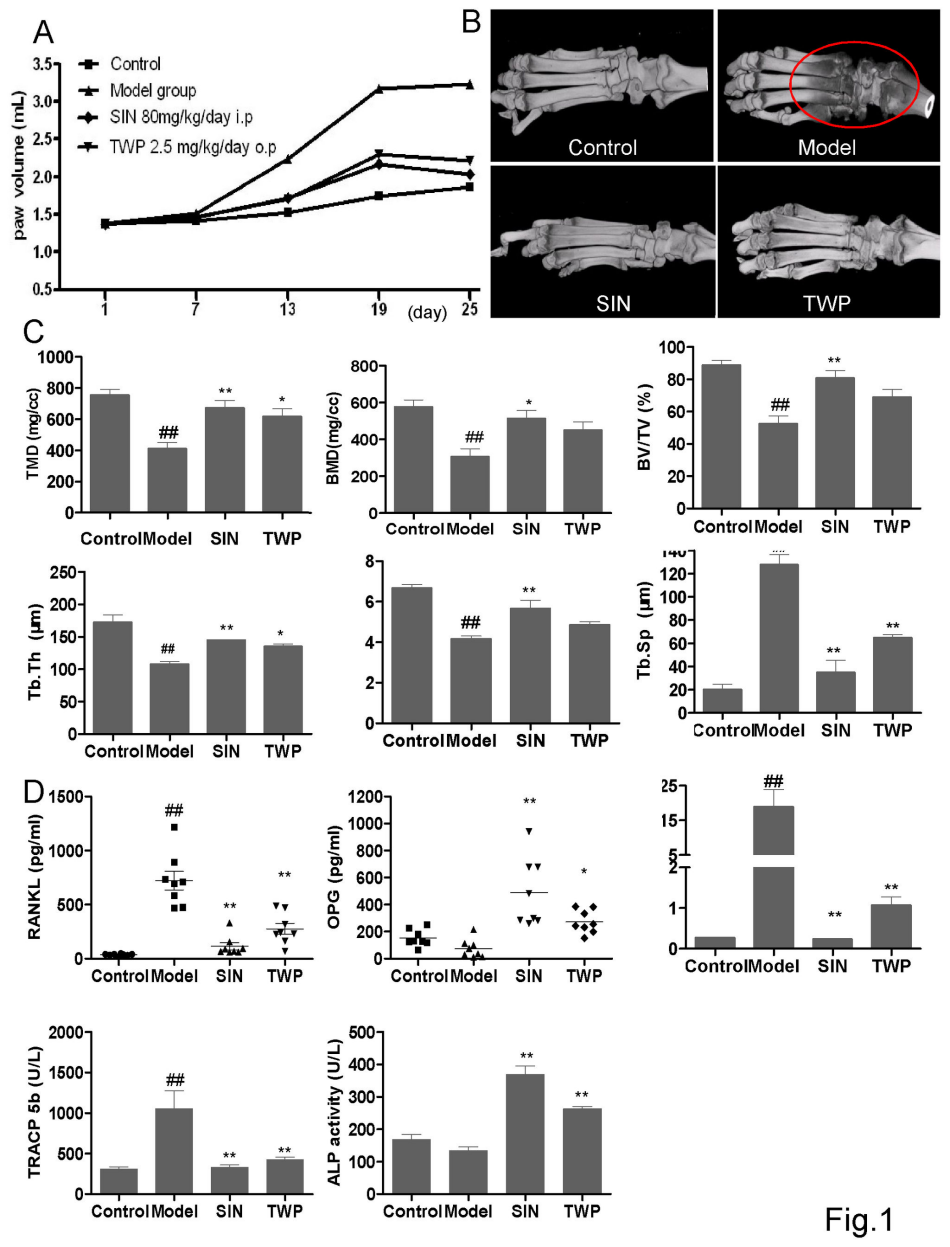
Sinomenine ameliorated Mt-induced arthritis and bone loss in rats. SD rat was injected with 2 mg heat-killed M. tuberculosis H37Ra (Mt) in 200 µl mineral oil subcutaneously at the base of the tail at day 0 to induce arthritis (n=8). SIN was administrated intraperitoneally at a dose of 80 mg/kg/day. Another anti-rheumatic drug, TWP, was administrated orally at a dose of 2.5 mg/kg/day. Rats in control group were not immunized with Mt. (A) Arthritic edema was indicated by measuring the swelling volume of the hind paw. At day 28, rats were killed and the hind lambs were stocked in 10% formalin to obtain (B) the 2-D image of hind lamb and (C) the bone parameters by Micro-CT. (D) Serum prepared from rat abdominal aorta blood was used to measure RANKL, OPG, RANKL/OPG ratio, TRACP5b (showing the osteoclast activity), ALP activity (showing the osteoblast activity). All the bars are mean±SEM of a representative experiment. *P<0.05; **P<0.01 (compared with model group); # P<0.05, # #P<0.01 (compared with control group).

Mt-induced arthritis model was used to evaluate the effects of SIN on osteoclast-mediated bone destruction *in vivo*. Bone loss was obvious around ankle joint in Mt-induced arthritic rats by micro-CT scan (Figure 1B). The bone density decreased in the circled area with the color changed from white to black, suggesting an increased osteoclast activity to resorpt bone in this animal model (Figure 1B). TWP ( *Tripterygium* *wilfordii* polyglycosides) is a well known Chinese drug for treating RA patients and arthritic animals [32], but it has many side effects such as causing menopause or sperm death, which prevents it from a long term use. As a positive control drug, TWP at 2.5 mg/kg/day obviously ameliorated the Mt-induced swelling and bone loss in rats (Figure 1). It was observed that SIN at 80 mg/kg/day reduced the hind paw swelling (Figure 1A) and bone loss (Figure 1B and 1C). The body weight increased after SIN treatment (data not shown), which is consistent with the previous studies [19,20]. For bone parameters, the tissue mineral density (TMD), bone mineral density (BMD), trabecular number (Tb. N), trabecular thickness (Tb. Th) reduced significantly in the arthritic model group when compared with the control group, while trabecular separation/spacing (Tb. Sp) increased dramatically. These changes of bone parameters are in consistence with bone loss, while SIN could obviously block these changes *in vivo* (Figure 1C and Figure S1). The images of bone resorption areas in different groups selected for bone parameter analysis are also included in Figure S1.

Since osteoclasts are bone-resorptive cells mediated by RANKL, we thus measured the serum TRACP5b and RANKL/OPG level. Matching with the attenuating effect on bone loss, SIN reduced serum TRACP5b and RANKL levels but dramatically increased the serum OPG level in arthritic rats resulting in the RANKL/OPG ratio close to the normal control (Figure 1D). The serum ALP activity was also upregulated significantly in SIN treated group (Figure 1D). Taken together, SIN blocked bone loss in Mt-induced arthritis in rats *in vivo*. These suggested that the suppression of bone loss by SIN might not only due to the inhibition of inflammation but also the reduction of osteoclast activity and RANKL related signals.

### Sinomenine inhibited RANKL-induced osteoclast formation *in Vitro*

**Figure 2:**
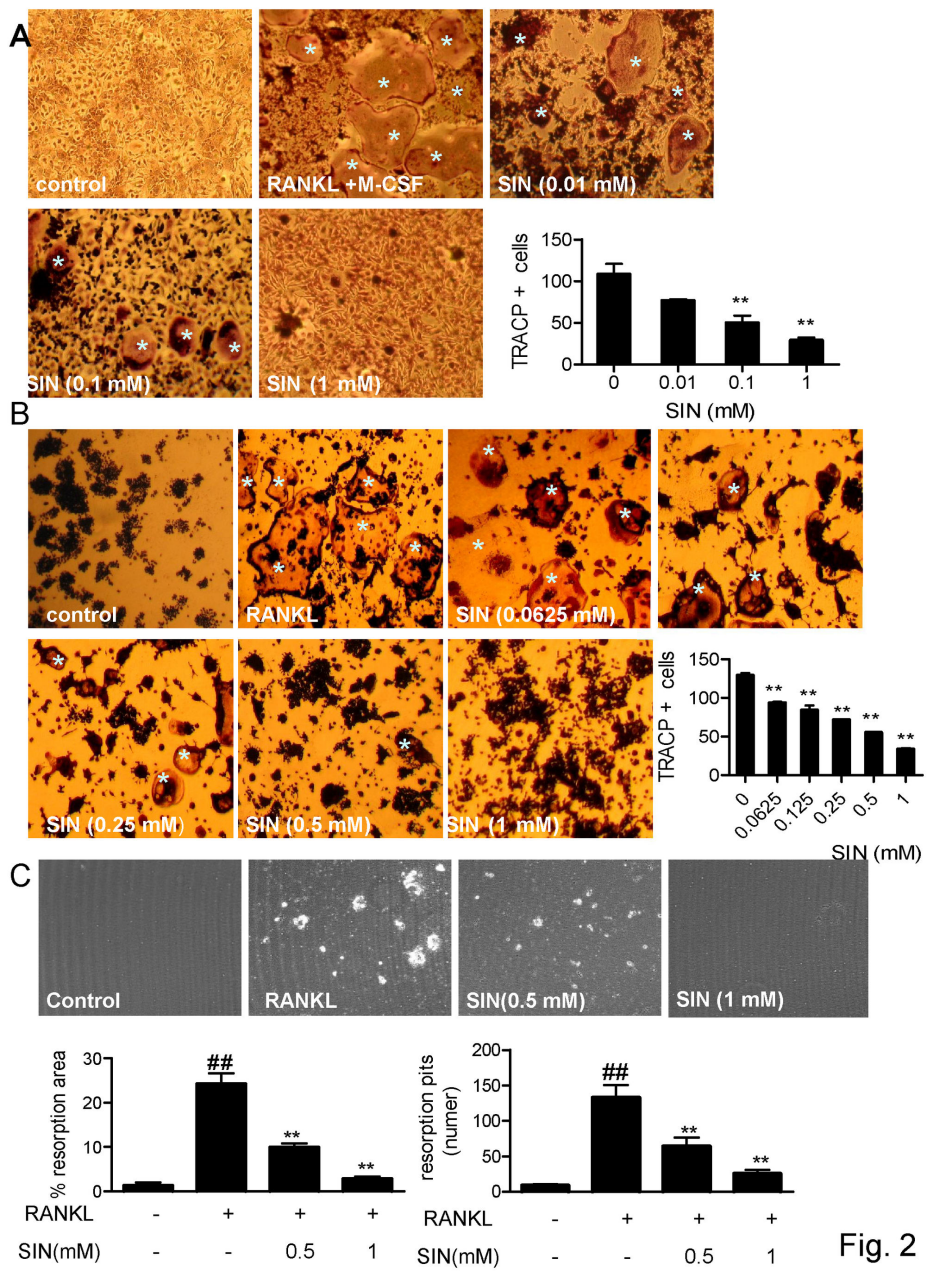
Sinomenine inhibited RANKL-induced osteoclast-like cell formation *in* *vitro*. (A) Primary bone marrow mononuclear cells were stimulated with RANKL+M-CSF (50 ng/ml) to evaluate the effect of SIN on osteoclasts formation by TRACP assay. (B) RAW264.7 cells stimulated by RANKL (100 ng/ml) were also used to value the effects of SIN on osteoclast formation and activity using TRACP assay and (C) bone resorption pit assay, respectively. TRACP^+^ osteoclasts were indicated by asterisks (*). All data are presented as mean±SEM of three representative experiments. *P<0.05; **P<0.01 (compared with RANKL+M-CSF group or RANKL group); # P<0.05, # #P<0.01 (compared with control group).

To address the direct effects of SIN on osteoclast formation, primary bone marrow mononuclear cells (BMM) from BALB/c mice stimulated with RANKL and M-CSF were investigated first. SIN inhibited the TRACP positive osteoclast-like cells (OCL) formation dose-dependently by reducing both the number and size of OCL. After treating with 1 mM SIN, the formation of large-size osteoclasts in the culture was abolished (Figure 2A). We then tested the drug effects on RANKL-induced osteoclast formation in RAW264.7 cells. As shown in Figure 2, SIN suppressed osteoclast differentiation dose -dependently from 0.0625 mM to 1 mM (Figure 2B). In addition, it reduced the RANKL-induced bone resorption at the concentrations of 0.5 and 1 mM (Figure 2C).

Cell viability assay revealed that SIN has no cytotoxic effects on RAW264.7 cells after treating at final concentrations of 0.5 mM or below for 48 h or 7 days by CCK-8 assay (Figure S2). SIN was observed to inhibit 60% of RAW264.7 cells proliferation at the high concentration of 1 mM only after a long time treatment such as 7 days in this study (Figure S2), which might be partly due to that the high concentration of SIN were reported to induce apoptosis in RAW264.7 cells [33].

### Sinomenine decreased the expression of RANKL stimulated osteoclastic specific genes and RANK/TRAF6

**Figure 3:**
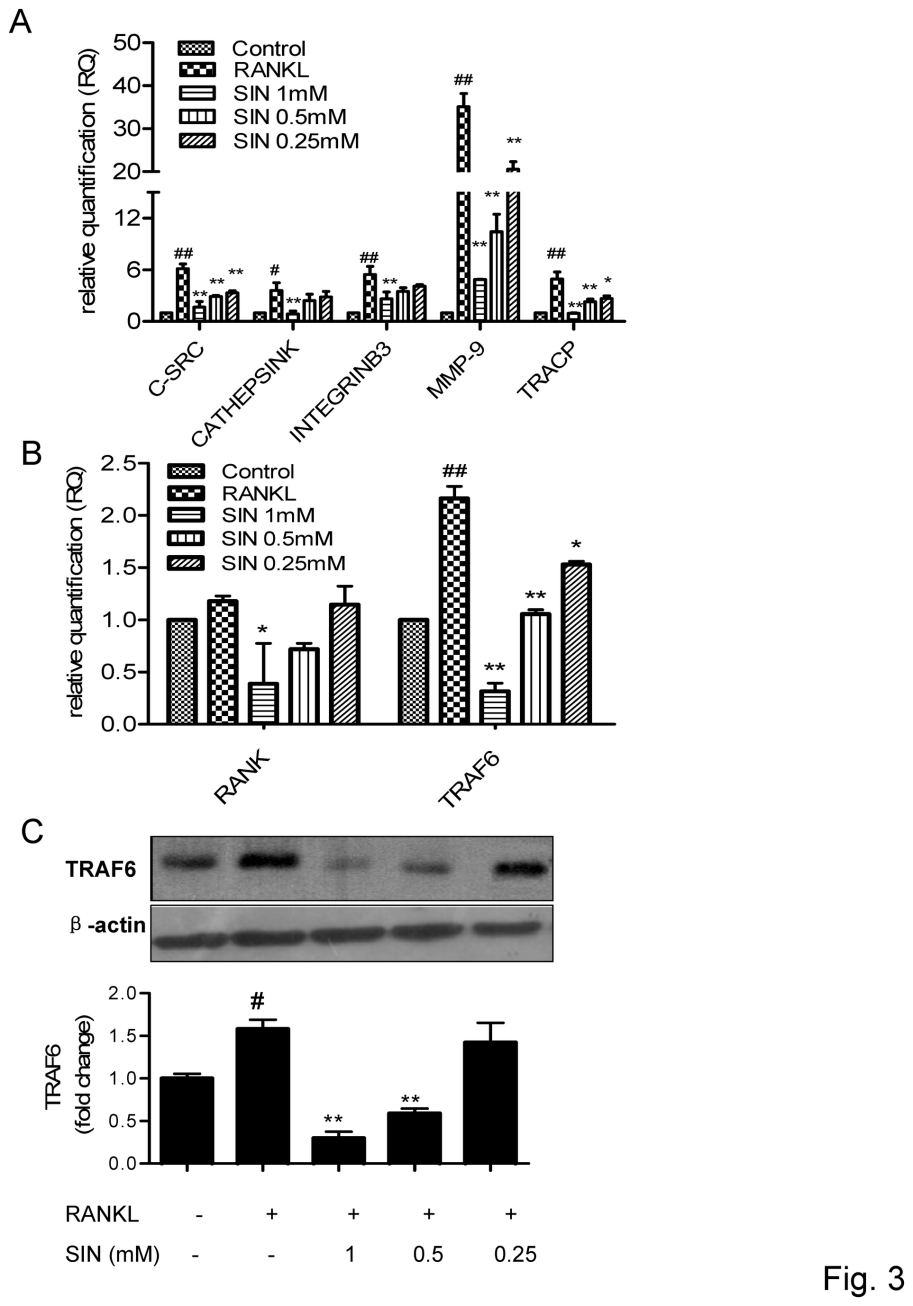
Sinomenine dose-dependently inhibited RANKL-induced osteoclast-specific genes and TRAF6. RAW264.7 cells were plated into 6-well plates, incubated with RANKL (100 ng/mL) and SIN for 24 h. Total RNA was extracted and real-time PCR was performed. Relative Quantiﬁcation (RQ) was used to determine the fold-change of gene expression compared with the GAPDH control gene. (A) osteoclast-specific genes, (B) RANK and TRAF6 genes expression were investigated. RAW264.7 cells were incubated with SIN for 30 min and then stimulated with RANKL for 30 min, (C) TRAF6 protein expression was analyzed by Western blot. β-actin was used as loading control. Densitometric quantification and statistical analysis include the results from 3 independent experiments. *P<0.05; **P<0.01(compared with RANKL group); # P<0.05, # #P<0.01 (compared with control group).

The marker genes required for osteoclast differentiation and bone resorption, including TRACP, cathepsin K, intergrin alpha-V/beta-3, MMP-9, c-Src were further examined by real time PCR. As shown in Figure 3A, SIN could obviously suppress the expression of osteoclast-specific genes after RANKL stimulation, especially c-Src, MMP-9, and TRACP, which were inhibited by SIN at a low dose.

TRAF6 has an essential role in osteoclastogenesis. In the initial step of RANKL-induced osteoclast formation, the recruitment of TRAF6 to RANK induces the TRAF6 trimerization, which leads to the activation of NF-κB and MAPKs [6,34]. Therefore, we evaluated the RANK and TRAF6 gene expression by real time PCR. The results showed that TRAF6 but not RANK mRNA were significantly increased after RANKL administration, SIN reduced the TRAF6 mRNA expression from the concentration of 0.25 mM obviously (Figure 3B). SIN could also inhibit the up-regulated TRAF6 protein from 0.25 mM by Western blot analysis (Figure 3C). Both the TRAF6 mRNA and protein were reduced even below the basal level when 1 mM SIN was added (Figure 3B-C)

### Sinomenine down-regulated RANKL induced NF-κB activation

**Figure 4:**
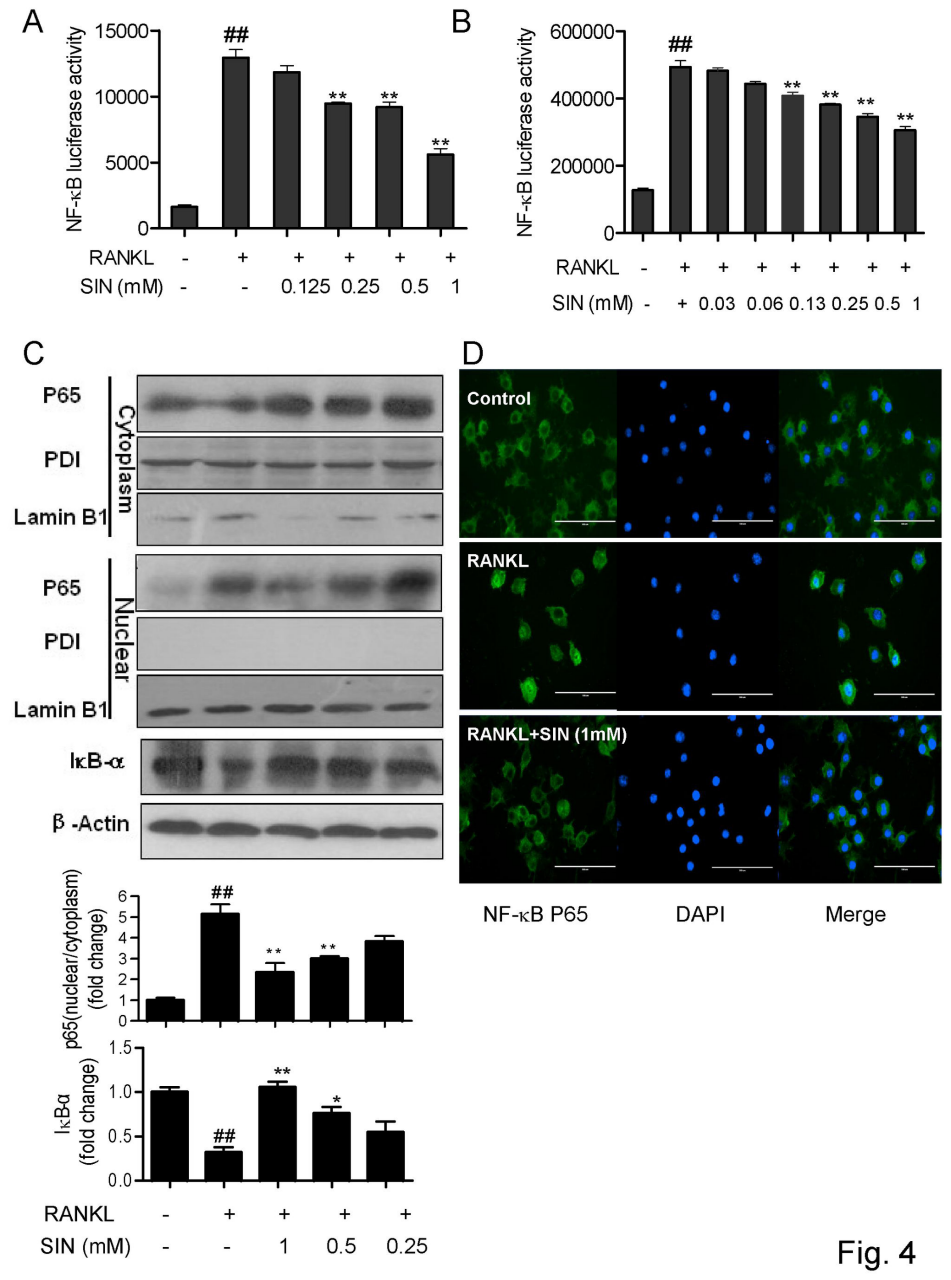
Sinomenine down-regulated RANKL-induced NF-κB **activation**. RAW264.7 cells, stably or transiently expressing NF-κB-luc reporter gene construct, were stimulated with RANKL for 8 h to study the effects of SIN on (A) stable or (B) transient NF-κB transcription. In another experiment, RAW264.7 cells were pretreated with SIN for 30 min and stimulated with 100 ng/ml RANKL for 30 min to (C) perform Western blot analysis of nuclear p65 and IκBα or (D) to observe p65 translocation to nuclear by fluorescence image. (C) The translocation of p65 by Western blot was expressed by dividing the nuclear p65 density with cytoplamic p65 density, the fold change of IκBα was normalized to β-actin. Densitometric quantification and statistical analysis include the results from 3 independent experiments. *P<0.05; **P<0.01 (compared with RANKL group); # P<0.05, # #P<0.01 (compared with control group).

TRAF6 trimerization leads to NF-κB activation during osteoclastogenesis. Three major steps are involved in the NF-κB activation, including the phosphorylation of IκBα, IκBα degradation and nuclear translocation of p50/p65. Gene transcription activity was evaluated in RAW264.7 cell line stably transfected with an NF-κB luciferase reporter construct. SIN from 0.25 to 1 mM reduced RANKL-induced NF-κB transcription significantly (Figure 4A). The results were also confirmed by the transient expression of NF-κB-luc in RAW264.7 cells. It showed that SIN from 0.25 to 1 mM dose-dependently decreased the NF-κB gene transcription induced by RANKL (Figure 4B, Figure S3A). Western blot further indicated that SIN inhibited IκBα degradation and p65 translocation to nuclear induced by RANKL (Figure 4C). The suppression of the nuclear translocation of p65 subunit was further confirmed by fluorescence microscopy (Figure 4D).

### Sinomenine modulated the phosphorylation of MAPKs induced by RANKL

**Figure 5:**
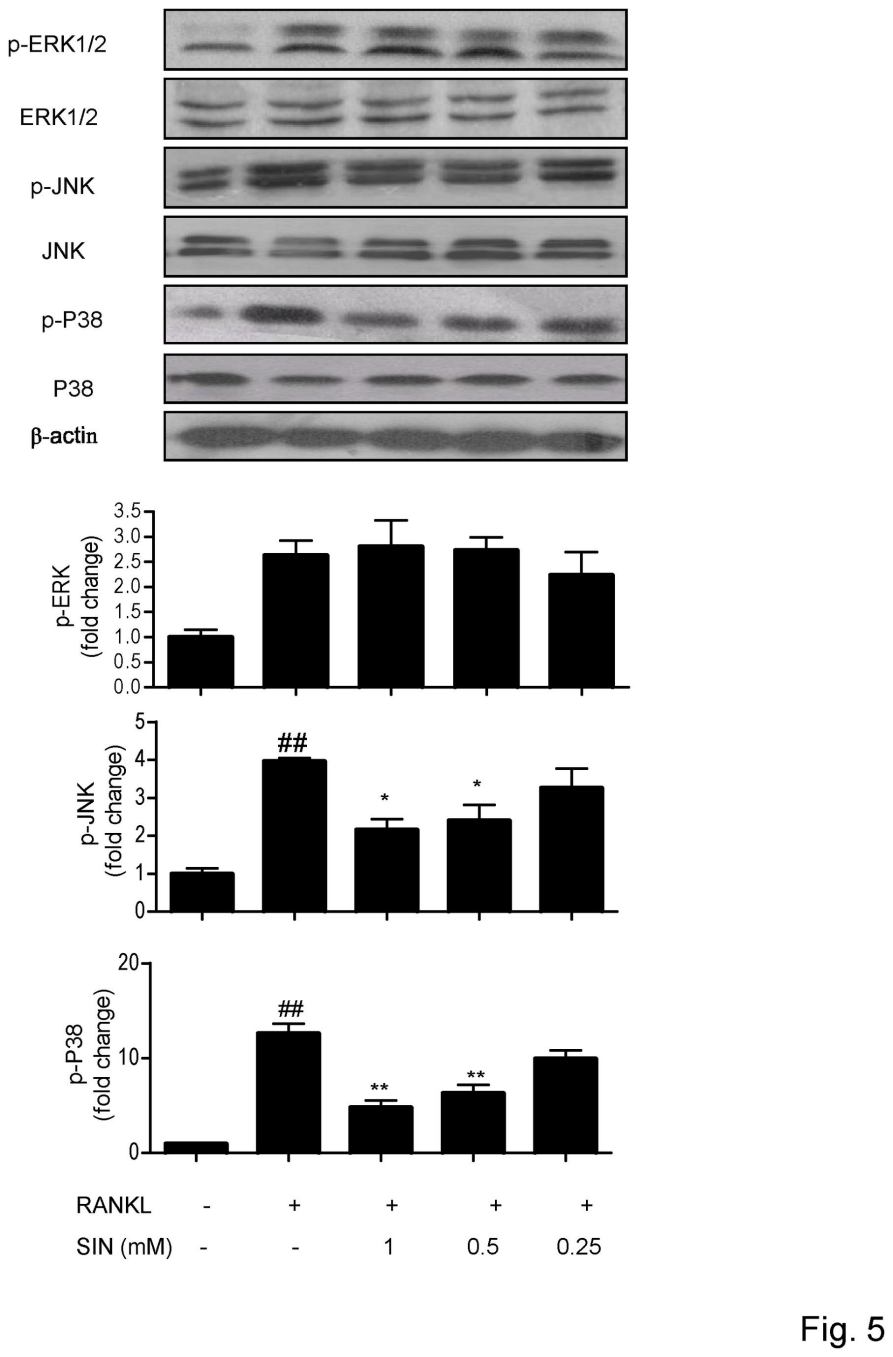
Sinomenine modulated RANKL-stimulated MAPKs. RAW264.7 cells were incubated with SIN for 30 min and then stimulated with RANKL for 30 min. Protein levels of phospho-MAPKs were detected by Western blot. ERK1/2, JNK and P38 were served as the loading control to p-ERK1/2, p-JNK and p-P38 respectively. Densitometric quantification and statistical analysis include the results from 3 independent experiments. *P<0.05; **P<0.01(compared with RANKL group); # P<0.05, # #P<0.01 (compared with control group).

MAPKs are also known to be activated after TRAF6 binding to its adaptor. Many *in vitro* experiments suggest that MAPKs play an important role in osteoclasts formation. MAPKs, such as p38, JNK and ERK1/2, are activated by RANKL during osteoclastogenesis. We found that SIN could obviously reduce the phosphorylation of p38 (p-p38) and JNK (p-JNK) in RANKL-stimulated RAW264.7 cells by Western blot (Figure 5). SIN did not decrease p-ERK1/2 responding to RANKL stimulation even at 1 mM (Figure 5)

### Sinomenine reduced RANKL-induced calcium influx

**Figure 6:**
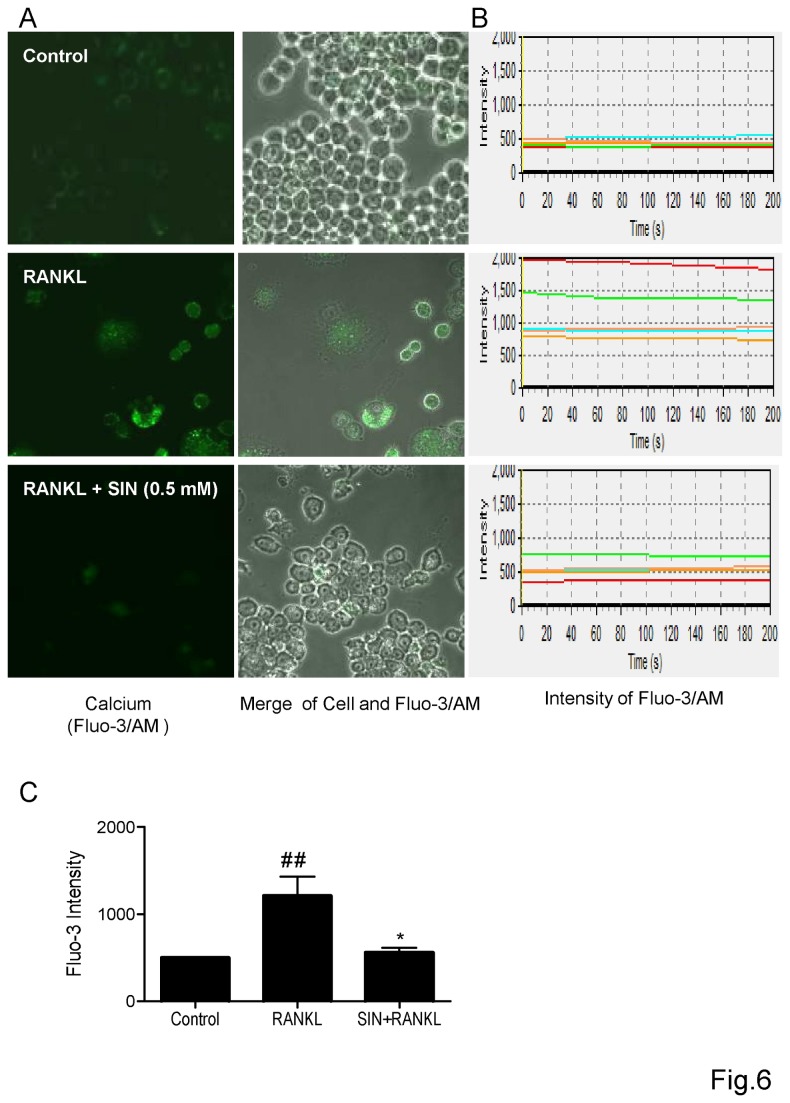
Sinomenine reduced intracellular calcium influx. RAW264.7 cells were incubated with RANKL (100 ng/ml) in the presence or absence of SIN (0.5 mM) for 72 h. For Ca^2+^ measurement, cells were incubated with Fluo-3/AM for 30 min in serum-free DMEM followed by confocal analysis. (A) Intracellular calcium was illustrated by green fluorescence (Fluo-3/AM). (B) Each line represented the fluorescence intensity of one cell. The fluorescence intensity in each group was recorded within 200s and (C) statistically analyzed at time point of 200s. P<0.05; **P<0.01 (compared with RANKL group); # P<0.05, # #P<0.01 (compared with control group).

Intracellular Ca^2+^ is a critical attribute to osteoclastogenesis and c-Src/TRAF6 complex is associated to the release of calcium [35,36]. Our results showed that SIN could inhibit c-Src and TRAF6 gene expression (Figure 3). Next, we determined if the downstream Ca^2+^ influx was affected by SIN in the pre-osteoclastic cells. RANKL could markedly induce an elevation of intracellular Ca^2+^ in RAW264.7 cells by measuring Fluo-3/AM fluorescence with confocal microscopy in 200s (Figure 6A). Our results showed that the treatment of SIN at 0.5 mM for 72 h could dramatically reduce the intracellular calcium in RANKL-stimulated cells, which exhibited by green fluorescence (Figure 6A). The fluorescence intensities of 5 individual cells in each group were recorded within 200s (Figure 6B) and statistically analyzed at time point of 200s (Figure 6C).

### Sinomenine inhibited RANKL-induced NFATc1 and AP-1 activation

**Figure 7:**
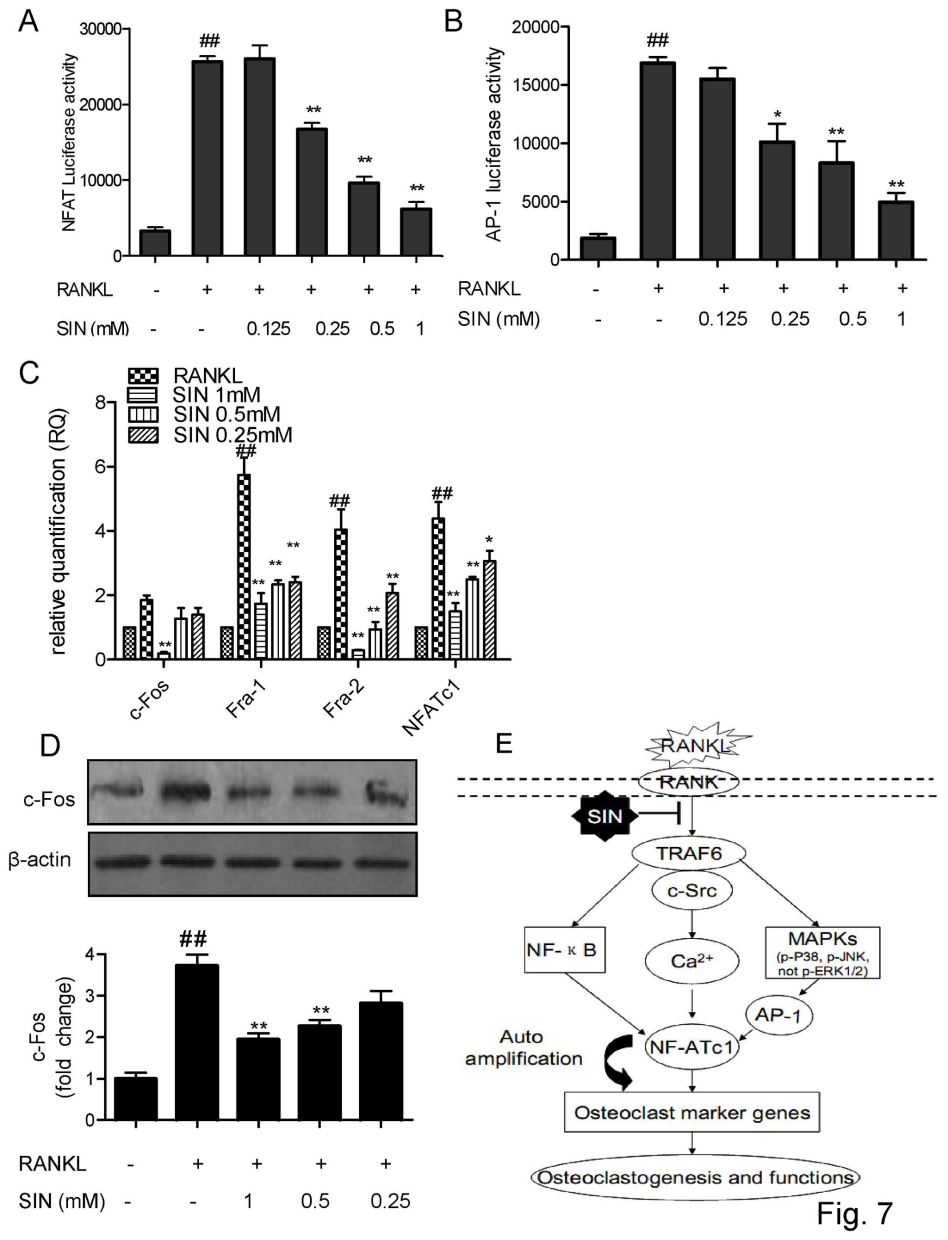
Sinomenine inhibited RANKL-induced NFAT, AP-1 activation. RAW264.7 cells, transiently expressing NFAT-luc or AP-1-luc reporter gene construct were pre-treated with SIN for 30 min and followed by 100 ng/ml RANKL stimulation for 12 h. (A) NFAT and (B) AP-1 transcriptions are indicated by luciferase activity. (C) RAW264.7 cells were plated into 6-well plates, incubated with RANKL (100 ng/mL) and SIN for 24 h. Real-time PCR was performed to determine gene expressions of NFATc1 and AP-1 components (c-Fos, Fra-1, Fra-2). RAW264.7 cells were incubated with SIN for 30 min and then stimulated with RANKL for 30 min. (D) c-Fos protein expression was analyzed by Western blot. β-actin was used as loading control. Densitometric quantification and statistical analysis include the results from 3 independent experiments. P<0.05; **P<0.01(compared with RANKL group); # P<0.05, # #P<0.01 (compared with control group). (E) A schematic diagram shows a hypothetical model by which SIN inhibits osteoclast differentiation and function.

NFATc1 is activated by Ca^2+^ signaling, and AP-1 components can be modulated by MAPKs. Because NFATc1 and AP-1 are also key transcription factors in RANKL-stimulated osteoclasts differentiation [6], it is interesting to see how these two pathways are affected by SIN. Transient transfection of reporter constructs showed that NFAT and AP-1 reporter gene expressions were dramatically up-regulated by RANKL stimulation, and down-regulated by SIN in RAW264.7 cells in a dose-dependent manner (Figure 7A-B, Figure S3B-C). By real time PCR, SIN was found to reduce NFATc1, and also AP-1 components (Fra-1 and Fra-2) genes expression dramatically from a low dose; while another important AP-1 component c-Fos was affected at a high dose (Figure 7C). Western blot analysis showed that SIN could decrease the RANKL stimulated c-Fos protein expression from 0.5 mM significantly (Figure 7D). Most of the osteoclastic specific genes are the target genes of NFATc1, so this suppression might in turn weaken the relative genes expression and osteoclast formation (Figure 7E).

## Discussion

Bone resorption by osteoclasts is a characteristic of many chronic inflammatory arthritic diseases, including RA. This process is frequently caused by excessive RANKL which has been validated as a useful target for treatment of pathological bone loss [3,10,29,37]. The currently used anti-RA drugs, such as methotrexate and dexemethone, are far from ideal to prevent bone destruction in RA patients. For example, both methotrexate and dexemethone are known to cause bone loss and promote osteoclast formation [38,39].

Sinomenine (SIN) has been used to treat RA patients with low side effects for several decades in China, while another Chinese herbal anti-RA drug TWP is limited in clinical use owing to its side effects in reproductive system despite its efficiency. Therefore it is intriguing to determine the role of SIN in osteoclast formation and bone resorption, and to elucidate its detailed mechanism of action.

In the present study, SIN was observed to reduce osteoclasts activity and bone resorption both *in vivo* and *in vitro*. Both bone loss and joint swelling could be blocked by SIN at a dosage of 80 mg/kg/day administrated intraperitoneally in Mt-induced arthritis in rats. Though the suppression of bone loss *in vivo* might be caused by the inhibition of inflammation and immune responses, the obvious intervenes of RANKL signals and osteoclast activity by SIN were observed. The serum TRACP5b and RANKL levels were decreased, while the serum OPG level was increased. It had been reported that the blockade of RANKL in the arthritic animals could abrogate joint damage despite the presence of joint inflammation [10]. Considering that the osteoclasts are bone-resorbing cells and RANKL is the key regulator of bone loss in inflammatory arthritis [40], the reduced osteoclast activity and RANKL, RANKL/OPG after SIN treatment might be beneficial to improve bone loss in arthritic rats. The inhibitory effects of SIN on osteoclasts and RANKL signaling were further investigated in RANKL stimulated pre-osteoclastic RAW264.7 cells. SIN inhibited TRAF6 and c-Src expression, disturbed several pivotal RANKL signal pathways including NF-κB, MAPKs/AP-1, NFATc1 during the ostoclastogenesis. These results suggest that the inhibitory effects of SIN on osteoclast differentiation and bone resorption *in vitro* might be partly due to the intervention of the excessive RANKL signals.

In the RANKL signaling pathways, NF-κB activation is essential in osteoclastogenesis and arthritis development [41,42]. SIN had been reported to inhibit NF-κB in human dendritic cells (DC) and macrophages/synoviocytes in arthritic rats [43,44]. In the present study, SIN attenuated the RANKL-induced NF-κB transcription activity, degradation of IκB-α, and the nuclear translocation of p65 in RAW264.7 cells. It could also decrease the mRNA expression of TRAF6 which is the upstream signal leading to NF-κB activation. This suggests that SIN acted on the early stage of osteoclast formation. Taken together, NF-κB activation seems to be a useful target mediating the inhibitory effects of SIN on osteoclastogenesis.

SIN could modulate RANKL-induced the phosphorylation of MAPKs which is downstream to TRAF6 trimerization. It reduced the phosphorylation of p38 and JNK, but it has little effect on the phosphorylation of ERK1/2 induced by RANKL. P38 and JNK are involved in osteoclast formation [6,45]. SIN had been shown to suppress activated p38 in DC or RBL-2H3 cells [43,46]. Thus, the suppressive effect of SIN on RANKL-induced osteoclastogenesis might be mediated partly by the suppression of activated p38 and JNK. On the other hand, though ERK1/2 is also activated by RANKL, ERK inhibitor does not suppress osteoclastogenesis but potentiates it [45,47]. Previous studies showed that 1 and 2 mM SIN induced apoptosis by activating ERK1/2 in normal RAW264.7 cells [33]. We also found that SIN at 1 mM seemed to increase the basal level of pERK1/2 (data not shown). Therefore, we assumed that SIN might both inhibit the RANKL up-regulated pERK1/2 by targeting upstream signals and activate the phosphorylation of ERK1/2 by other unknown mechanisms, which could result in no significant effect on pERK1/2 overall. The mechanism by which SIN acts on ERK1/2 to affect the generation and survival of osteoclasts deserves further investigation in the future.

MAPKs activate transcription factor AP-1 [48]. SIN could also inhibit RANKL-induced AP-1-luc transcription and the gene expression of AP-1 components such as Fra-1, Fra-2, c-Fos. Fra-1 can rescue the osteopetrosis (lacking osteoclasts) in c-fos-mutant mice *in vivo* [49], and Fra-2 controls osteoclast survival and size [50], which might explain the attenuating effect of SIN on osteoclastogenesis.

NFATc1 is the major transcription factor mediated by activated calcium signal, while the TRAF6/c-Src complex is involved in releasing intracellular Ca^2+^ in osteoclastogenesis [29,36]. We first found that c-Src and TRAF6 genes were dramatically inhibited by SIN. In addition, SIN was previously observed to decrease intracellular Ca^2+^ in cultured rabbit aortic vascular smooth muscle cells (VSMC) [51-53], decrease calcium ionophore-stimulated prostaglandin E2 (PGE_2_) and leukotriene C4 synthesis in RAW264.7 cells [54]. We also observed that SIN abrogated the calcium ionophore inomycin-induced NFATc1 transcription in an NFAT-luc gene stably transfected Jurkat T cell line (data not shown). Therefore, we postulated that SIN might also disturb the regulation of Ca^2+^ and NFATc1 in response to RANKL during osteoclastogenesis. Our finding demonstrated for the first time that SIN could decrease the intracellular Ca^2+^, and inhibit NFATc1 mRNA expression and transcription activity in RANKL stimulated pre-osteoclastic RAW264.7 cells. Since NF-κB and AP-1 can bind to NFATc1 promoter and NFATc1 can regulate most osteoclast maker genes, SIN might inhibit NFATc1 to suppress the osteoclast-specific genes such as TRACP, c-Src and MMP-9, and RANKL-induced osteoclast formation.

Taken together, SIN reduced osteoclast formation and attenuated the majority of RANKL/RANK/TRAF6 mediated signals during osteoclastogenesis. In addition, SIN might also affect the upstream of RANKL given that the serum levels of RANKL were decreased. Moreover, SIN is known to block the activation of NF-κB [43] and the direct binding between an active SIN derivative and NF-κB (P50) have been computer-aid modeled [55]. Such molecular activity by SIN might affect NF-κB translocation, transcription and gene expression. In conclusion, considering anti-RANKL and anti-osteoclast therapies have shown to be beneficial for bone loss in arthritis patients in clinical trial [34], these results provided a new insight that sinomenine might be useful for the treatment of RA patients partly via attenuating osteoclast formation and RANKL related signaling pathways.

## Supporting Information

Figure S1 **Mico-CT images for bone parameter analysis in rats.** SD rat was injected with 2 mg heat-killed M. tuberculosis H37Ra (Mt) in 200 µl mineral oil subcutaneously at the base of the tail at day 0 to induce arthritis (n=8). SIN was administrated intraperitoneally at a dose of 80 mg/kg/day. Another anti-rheumatic drug, TWP, was administrated orally at a dose of 2.5 mg/kg/day. Rats in control group were not immunized with Mt. The circled areas indicated the position for the bone parameters analysis in Figure 1C within ankle joint of each group by micro-CT.
(TIF)

Figure S2 **Effects of SIN on the RAW264.7 cell viability.** The experiment was performed by a CCK-8 assay at 48 h and 7 days post treatment as described in Method. All bars are mean±SEM of representative of 3 independent experiments. The significance was determined by ANOVA-test. *P<0.05, **P<0.01, as compared with a control group.
(TIF)

Figure S3 **Effects of SIN on transient NF-κB, NFAT or AP-1 expression by the dual-luciferase reporter assay.** To examine NF-κB, NFAT or AP-1 activation, RAW264.7 cells were seeded in 96-well plates at a density of 1×10^4^ cells/well and incubated for 24 h. The cells were then transfected with 0.2 µg of the NF-κB, AP-1 or NFAT luciferase reporter constructs (respectively) and 0.2 µg of Renilla luciferase control vector according to the manufacturer’s instructions. Turbofect (Fermentas) was used for the transfection. Empty pGL-TK vector (Promega) was used as control. After 48 h, medium was changed with complete DMEM and pre-treated with SIN for 30 min followed by adding RANKL (100 ng/ml) for 8 h, the cells were then harvested and analyzed by the Dual-luciferase Reporter Assay System (Promega) according to the manufacturer’s instructions. Data were normalized for transfection efﬁciency to Renilla luciferase activity and relative luciferase units (RLU) of (A) NF-κB, (B) NFAT or (C) AP-1 were calculated as firefly luciferase activity/Renilla luciferase activity. All bars are mean±SEM of triplicate. The significance was determined by ANOVA-test. *P<0.05; **P<0.01 (compared with RANKL group); #P<0.05, # #P<0.01 (compared with control group).
(TIF)
