# Supplementary figures and images for: Sinomenine Suppresses Osteoclast Formation and Mycobacterium tuberculosis H37Ra-Induced Bone Loss by Modulating RANKL Signaling Pathways
Source: PLoS One. 2013 Sep 16;8(9):e74274. doi: 10.1371/journal.pone.0074274 (PMC3774760; doi:10.1371/journal.pone.0074274)

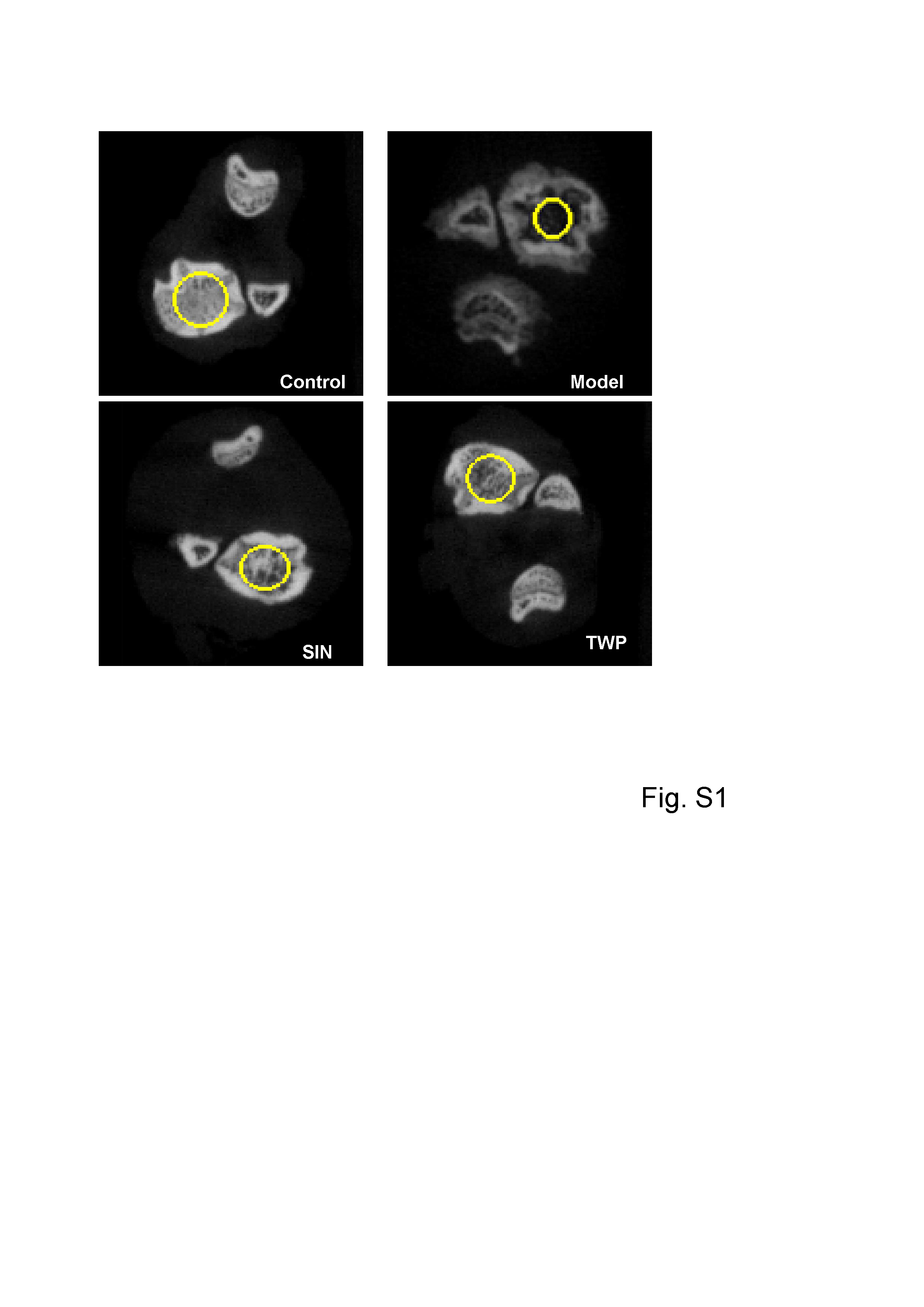

Supplement: Figure S1 — Mico-CT images for bone parameter analysis in rats. SD rat was injected with 2 mg heat-killed M. tuberculosis H37Ra (Mt) in 200 µl mineral oil subcutaneously at the base of the tail at day 0 to induce arthritis (n=8). SIN was administrated intraperitoneally at a dose of 80 mg/kg/day. Another anti-rheumatic drug, TWP, was administrated orally at a dose of 2.5 mg/kg/day. Rats in control group were not immunized with Mt. The circled areas indicated the position for the bone parameters analysis in Figure 1C within ankle joint of each group by micro-CT. (TIF) [file pone.0074274.s001.tif]

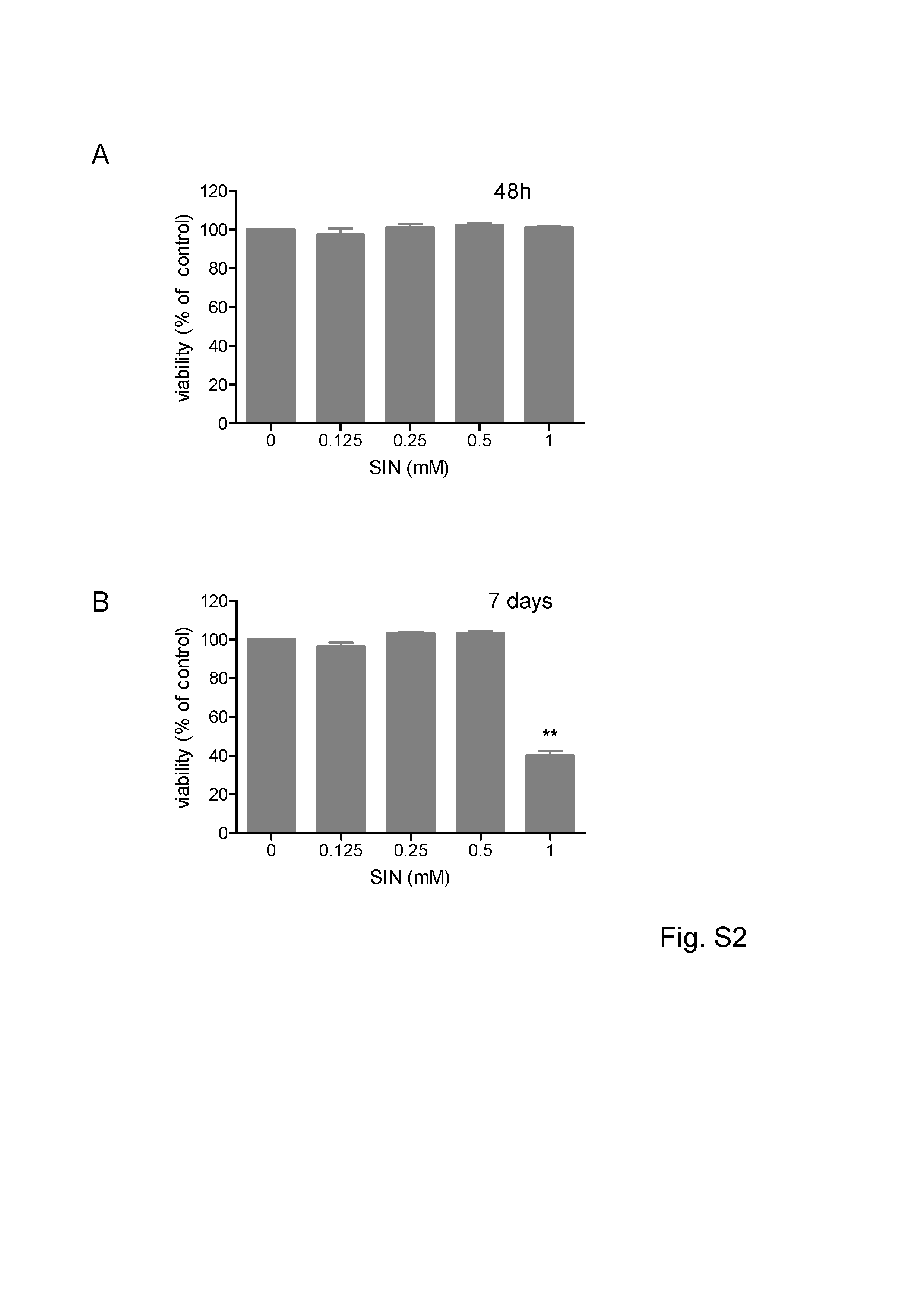

Supplement: Figure S2 — Effects of SIN on the RAW264.7 cell viability. The experiment was performed by a CCK-8 assay at 48 h and 7 days post treatment as described in Method. All bars are mean±SEM of representative of 3 independent experiments. The significance was determined by ANOVA-test. *P<0.05, **P<0.01, as compared with a control group. (TIF) [file pone.0074274.s002.tif]

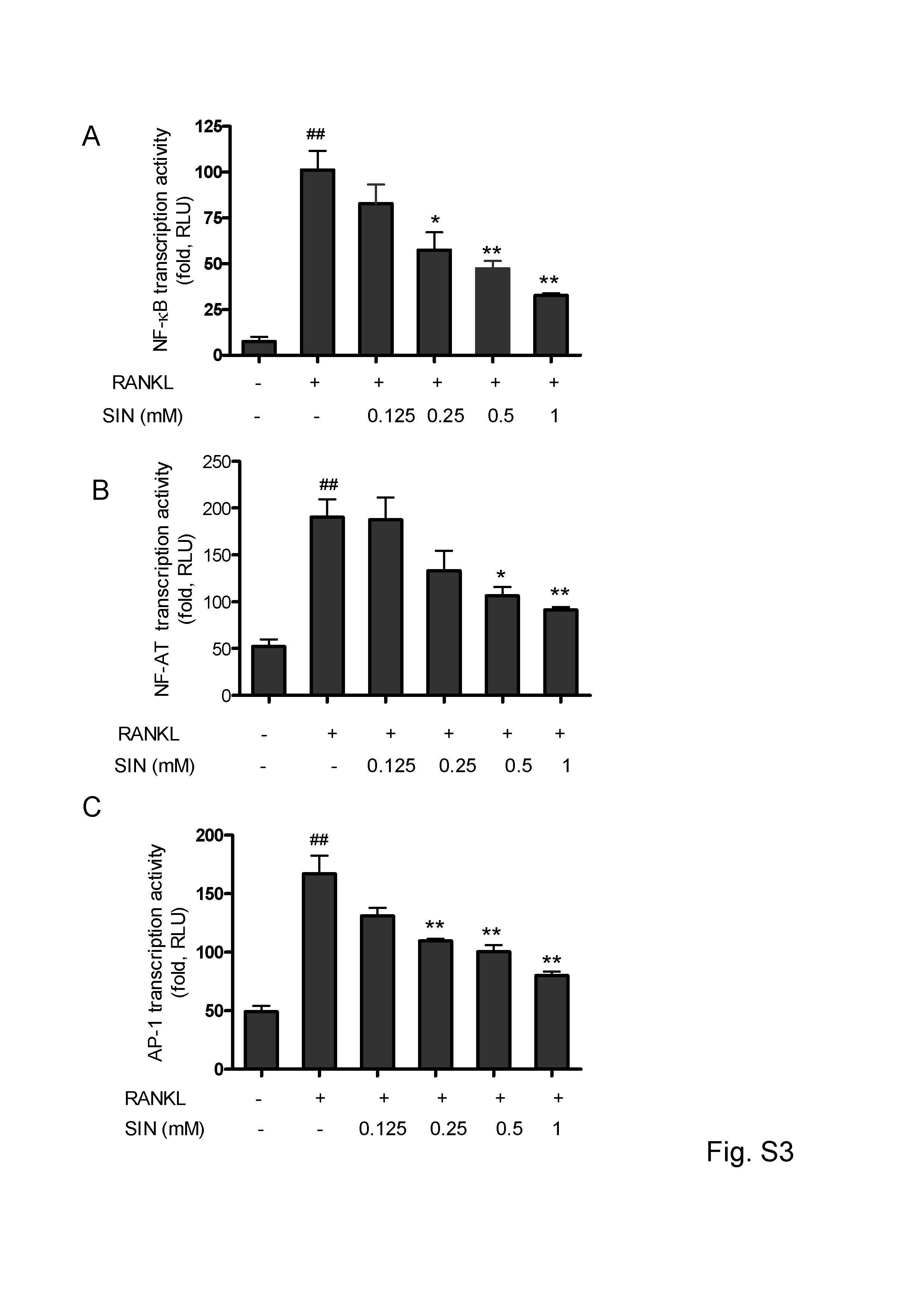

Supplement: Figure S3 — Effects of SIN on transient NF-κB, NFAT or AP-1 expression by the dual-luciferase reporter assay. To examine NF-κB, NFAT or AP-1 activation, RAW264.7 cells were seeded in 96-well plates at a density of 1×10^4^ cells/well and incubated for 24 h. The cells were then transfected with 0.2 µg of the NF-κB, AP-1 or NFAT luciferase reporter constructs (respectively) and 0.2 µg of Renilla luciferase control vector according to the manufacturer’s instructions. Turbofect (Fermentas) was used for the transfection. Empty pGL-TK vector (Promega) was used as control. After 48 h, medium was changed with complete DMEM and pre-treated with SIN for 30 min followed by adding RANKL (100 ng/ml) for 8 h, the cells were then harvested and analyzed by the Dual-luciferase Reporter Assay System (Promega) according to the manufacturer’s instructions. Data were normalized for transfection efﬁciency to Renilla luciferase activity and relative luciferase units (RLU) of (A) NF-κB, (B) NFAT or (C) AP-1 were calculated as firefly luciferase activity/Renilla luciferase activity. All bars are mean±SEM of triplicate. The significance was determined by ANOVA-test. *P<0.05; **P<0.01 (compared with RANKL group); #P<0.05, # #P<0.01 (compared with control group). (TIF) [file pone.0074274.s003.tif]
